# A Case of Protrusion of the Spleen through an Opening in the Abdomen

**Published:** 1872-10

**Authors:** W. H. Williams

**Affiliations:** Shreveport, La.


ART. II.—*A case of Protrusion of the Spleen through an opening made into the Abdomen, with a Pocket Knife.* By W. H. WILLIAMS, M. D., Shreveport, La.

John Beech a Freedman, 21 years of age, was admitted into the Infirmary Dec. 24th, 1868. He stated that whilst serving as a deck-hand on the steamboat Bart Able. He got into an altercation with another hand, who stabbed him in the left side, and that he thought his entrails were coming out through the opening. He stated that he was stabbed the morning of the day before, making it about twenty-eight hours before he could get medical aid. He had some trouble in getting admittance into the Infirmary which forced him to walk fully one mile after leaving the boat. He was very much exhausted, quite bloody, and still bleeding. His respiration was short and hurried, pulse weak and quick, skin cadaverous in appearance and cold to the touch, tongue almost white; in short he showed evidences of great suffering, and much loss of blood.

On examination a mass the size of a walnut, was formed protruding through and partly strictured by a very small opening, at the anterior, and lower margin of the spleen. This mass was quite black with a very smooth surface, and blood was oozing from it all the time. The opening through the skin and integuments, transversly to the trunk, was so small, and the protruding mass so hard, we could not return it into the cavity, and besides we did not think it best to do so, not having satisfied ourselves beyond a doubt as to its nature. We ordered him bandaged with the view of preventing further enlargement of the hernial mass, and ordered two grs. of Pulv. Opii in a stiff toddy, and to repeat opium and toddy every four hours until reaction is established.

Fourteen hours after, the only material change that had taken place was an enlargement of the hernial mass, which was about twice as large as when first seen by us.

We were now satisfied that it was spleen substance that we had to deal with, and that it would not do to cut it off and return it to the cavity of the abdomen.

The expedient resorted to was to pass a silver wire through the integument and neck of the hernial mass, transversely to the cut, returning the wire to the side of entrance, so as to completely, if possible, strangulate the tumor between the edges of the wound. The distance between the points of entrance and exit of the wire, on each side, was about one-third of an inch. After tightening the wire and fastening it with a perforated shot, it was still apparent that it continued to bleed. We then passed a ligature around the neck of the mass sufficiently tight to stop the oozing of the blood, but not tight enough to hurry the sloughing off of the part. This I regard as the most important point adopted in the management of the case, and that which led to his recovery.

Had the neck of the mass been strangulated suddenly that portion of the spleen internal to the wound would have fallen back into its old position, and owing to the great vascularity of the spleen, would have very soon bled the man to death.

I will state that it was one month from the time the wire was introduced until it was removed, and that then the mass external was not quite even with the surface of the body, and even now, two months after he was stabbed, the wound is not entirely well, yet the boy is at work every day.

The general treatment adopted in this case was purely paliative, with a strict regard to regimen and the recumbent position.

He was kept in bed five weeks, during which period it was necessary most of the time to keep him under the influence of an opiate to relieve pain.

I omitted to state that he had two or three spells of vomiting when he was first admitted that gave him great pain. He had very little heat of the skin at any time, and he was forced to take nourishment up to the third week.

He stated that he had not been subject to chills, and that he had always been healthy, and a hard worker. This being assumed as a fact, I am free to say that he lost at least half of the spleen, and I now believe him perfectly safe, and I might say well.

